# Medical News and Miscellany

**Published:** 1897-11

**Authors:** 


### Medical News and Miscellany.

### Organization of the Texas Medical Profession.

*To the Medical Profession of Texas:*

The old committee on Medical Societies, of the Texas State Medical Association, has been re-appointed; the work expected of this committee being the organization of the medical profesof Texas in the best manner possible.

With a feeling of diffidence, we again come before you to present some of the claims which the noble profession of medicine has upon you, also to point out some of the needs of the profession in this State and to suggest a plan of organization and work by which we hope to accomplish material results and lasting benefits.

Too many physicians go through life believing their duty has been fully discharged when they have completed their daily business satisfactory to themselves, neglecting their duty towards those who have brought the profession to its present high standard of excellence.

It is the unquestionable duty of every regular graduate in medicine to belong to a local and State medical association. If there is no local society in his community he should see to it that one is organized; if not in his county, then in his district. The physician owes it to his patrons, to his profession and to himself to affiliate with and to work for the cause of legitimate medicine. The medical society is educational. By a mutual interchange of ideas at the society meetings the most stupid can gain something and the most learned may add to his knowledge. Our most intelligent and successful physicians are medical society men. There is nothing else which so broadens a man's views and increases his interest in his chosen profession as free intercourse with his fellows, and this is especially applicable to the medical profession. The foregoing being true it is clearly the duty and the privilege of the physician to benefit himself and his patrons by attending the medical society meetings. Not only may we and our patients be benefitted by our attendance on medical society meetings, but through these organizations, and united effort, the standard of medicine may be elevated and efficient laws for the protection of the people and the profession may be secured.

A MEDICAL LAW.

For twenty years the profession of Texas has been burdened with a crazy-quilt medical law, the provisions of which are almost totally inoperative. It stands on the statute books a mere pretense, and is a disgrace to the great State of Texas and her grand army of noble medical men. Through the inefficiency of this law our state has been brought into disrepute, the impression being general that "any one can practice medicine in Texas," consequently our State has been deluged with the incompetents, ignorant pretenders and blatant quacks from other states; and by undergraduates who hold a certificate from a "district board" of our own State. Texas is a rich field for these ignorant pretenders.

When the last legislature was asked to enact a medical law, which was framed and favored by the Texas State Medical Association, it refused, and instead, it gave us the iniquitous and unjust

OCCUPATION TAX.

One of the medical journals of Texas has very aptly said, "The medical profession of Texas asked for bread and was given a stone." The many noble deeds of charity done by physicians

(more than all other professions and trades combined) counted for naught, the State, through her law makers, saw a chance to raise revenues by unjustly taxing a class of her most charitable citizens who were not well enough organized to resist such unjust measures.

Another matter that deserves the earnest consideration of the medical profession of this State is the establishment of a

STATE BOARD OF HEALTH.

Nearly all of the more progressive states of the Union have a state board of health, and these states have found them not only useful but an actual necessity.

To elevate the standard of medicine, to secure laws protective alike to the profession and the people, such as will advance our common interests, our only hope is in organization. We appeal to every regular graduate in medicine to use every effort to increase the membership in medical societies where they exist, and to effect their organization where, at present, there are none. Make of it a personal matter, talk "medical society" to your brother physicians, arouse their interest and enthusiasm until every eligible physician is a member of at least a local society. Those who organize city, county, and district medical societies should, and we believe will, be gratefully remembered by the other members of the medical fraternity, too much cannot be said in their praise.

At the meetings of the local society is the proper place to present the claims of the State Association, and we, therefore, call upon the secretaries of the different local societies to provide space on their programs for inviting members to join the State Association.

Let every man do his whole duty and within a few years we will have secured a medical organization so perfect in its completeness and so powerful in its influence for the good of humanity that any and all physicians will be proud to be numbered with the Texas Medical profession.

Respectfully,

S. E. Hudson, Austin, Chairman,
L. L. Shropshire, San Antonio,
J. D. Becton, Greenville,
W. R. Blalock, McGregor,
E. A. Woldert, Tyler, Secretary.
Committee.

**Dr. J. Q. Burton,** has removed from Mt. Selman to Troupe, Texas.

---

**The** National Prison Association will meet in Austin, Texas, December 2 to 6 prox.

---

**Dr. Jo Ralston,** a young Austinite, recent graduate of Texas Medical College, is quarantine officer at Bolivar Point, off Galveston.

---

**Dr. Russell Caffery,** long time a leading physician of San Antonio, has removed to Houston. His office is Nos. 327–328 Binz building; residence, 1110 Caroline street.

---

**Dr. John R. Frazier,** of Monterey, Mexico, has located at Belton, Texas, and formed a co-partnership with his brother, Dr. J. M. Frazier of that place.

---

**Dr. Henry Leath** and his wife have the sincere sympathy of the JOURNAL in the death of their only daughter, Inez, which occurred at their home in Quitman, Texas, October 18, ult. She was five years of age.

---

**Dr. Bucke,** of the Insane Asylum, at London, Ont., says that dogs and monkeys possess the senses of shame and remorse and even of the ludricious. We have often heard it said of certain things, "enough to make a dog laugh."

---

**The Journal** regrets to learn that a painful accident befell the little daughter of Dr. W. P. Powell, of Willis, recently. She fell out of a tree, sustaining serious injuries, for which she has been taken to Houston for treatment.

---

**Good Country Practice,** with comfortable house, outbuildings, etc., for sale at a sacrifice. Good reasons for wanting to sell. A small cash payment and balance on time. Address Dr. G. M., care of TEXAS MEDICAL JOURNAL.

---

**The Congress of the National Prison Association** which was to have been held in Austin, Texas, October 16th, ult., was postponed on account of yellow fever in the South, until December 2 to 6. A large attendance is expected. We shall have with us on that occasion some of the most distinguished men in America; jurists, physicians, advocates, wardens, asylum superintendents, chaplains and a few plain folks.

---

**Postponed:** The meeting of the Tri-State Medical Association which was to have taken place at Memphis, November 17 and 18, inst., is postponed till December 15 and 16, prox., on account of yellow fever. Dr. Richmond McKinney, the editor of the *Memphis Medical Journal* is secretary.

---

**The Southern Surgical and Gynecological Association.**—The tenth annual meeting of this association will be held at the Southern Hotel, St. Louis, Mo., on November 9, 10 and 11, 1897. Members of the medical professional are cordially invited to attend. A very full and interesting program has been arranged.

---

**An Application for Chloasma.**—The *Centralblatt fur die gesammte Therapie* for October credits the following to the *Deutsche Medicinische Wochenschrift:*

℞ Chloral hydrate........................................ 2 parts;
Carbolic acid,
Tincture of iodine, } each..... ...................... 1 part.

M. To be applied with a brush.

---

**"Empyaema."**—We sympathize with our learned English contemporary the *Lancet* in its regret at the gradual disappearance of the diphthong from words adopted from the Latin or Greek. We think it should be retained in technical words. But we fear that the *Lancet* has fallen into error in one of its illustrations of the change of spelling. In its issue for September 25th it says: " 'Empyema' is now never written 'empyæma.' " Dear contemporary, we do not know that it ever was.—*New York Medical Journal.*

---

**A New Story by Lydston.**—Every doctor who has heard Dr. G. Frank Lydston, of Chicago, tell one of his stories in his inimitable way, or has read "Over the Hookah," will be glad to learn that a new story by Lydston will soon appear in the *Tri-State Medical Journal*, of St. Louis. It is entitled: "The Doctor's Crœsus—the Tale of a Generous Patient." It will be of great interest and is to be fully illustrated. Physicians who are not already subscribers to the *Tri-State Medical Journal* can obtain the numbers containing Dr. Lydston's latest effort by sending 25 cents to the business manager, 3509 Franklin Avenue, St. Louis, Mo.

---

**An Application for Pigmentary Blemishes of the Skin.**—The *Journal de Medicine de Paris* for September 26th gives the following formula:

| | | | |
|---|---|---|---|
| ℞ | Corrosive sublimate............................ | $7\frac{1}{2}$ | grains; |
| | White sugar.................................... | 225 | " |
| | The white of one egg; | | |
| | Lemon juice..............................about | 450 | " |
| | Distilled water.................................. | 3,750 | " |

M. S.: To be applied every morning and allowed to dry on.

---

**Kind Words.**—"The 'Red Back' is one of the essentials at my 'shop,' and as long as I am in business will keep it in stock. Wishing you all the prosperity possible, I am yours truly,

"R. R. Shapard."

"Franklin, Texas, Oct. 6, 1897.

"Drs. Daniel and Hudson:

"Esteemed Sirs:—

"I don't remember when I remitted last, as I generally send $5 at one time for the 'Red Back;' so, for fear I am nearing the limit of my last remittance, I hereby enclose $5, to be placed to my credit. I never expect to do without the 'Red Back' while I practice medicine, and if I quit the practice I expect to take it anyhow. Yours sincerely,

"G. M. Abney."

A great many subscribers in renewing their subscriptions say nice and pleasant things, like the above, all of which is well appreciated.

---

**Pyrethrum as a Remedy.**—Dr. G. R. Plummer, of Key West, Florida, writes to the editor of the *Journal of the American Medical Association* asking his professional brethren to experiment with insect powder, the flowers of *Pyrethrum carneum* and *Pyrethrum roseum*, which plants, he says, are now largely cultivated in California. In consequence of an accident to a child, says Dr. Plummer, it seems probable that pyrethrum has anthelminthic properties, and he would like to know what doses of it are proper for man. As it is frequently adulterated, he asks experimenters to take pains to get a perfectly pure article.—*Ex.*

---

**An Intestinal Antiseptic Mixture.**—According to the *Independance Medicale* for September 29th, the following formula is advised by de Maximovitch:

| | | |
|---|---|---|
| ℞ | Naphthol........................................ | 45 grains; |
| | Chloroform.................................... | 15 drops; |
| | Castor oil........................................ | 1,500 grains; |
| | Essence of peppermint........................ | 5 drops. |

M. Dose, a tablespoonful (for children, a teaspoonful) in port wine, beer, or hot and sweetened black coffee.

---

**To Fill a Long-Felt Want.**—We are in receipt of a prospectus for "a weekly paper for the people of Texas," to be called *The Alcalde.* We extract from it the following:

"There appears to be a well-defined opportunity to do genuine service for a weekly paper of the character we shall emulate. One department, from which great results are hoped for, will constitute an absolutely unprejudiced forum for occasional correspondents. It is expected that many a thoughtful man, devoted to the general welfare, will come to appreciate this means of realizing himself, of exerting his influence for the public good, unhampered by considerations of local prudence on the part of daily papers. Too often such men have discovered that powerful local interests render discussion of some questions inexpedient. This is stated as a simple fact, and not at all in animadversions against daily papers; but only to make clear that there is a distinct office for a general state weekly. Each has its sphere. There is room for both."

That's it; if any paper has the backbone or the "wherewith" to buck against "powerful local interests," it will fill a long-felt want and—an early grave, we are sorry to add. We want to see if *The Alcalde* will tackle the saloon, the whiskey traffic, the patent medicine and other nostrum nuisances; and will shut its pages to Dr. Hartman, Lydia Pinkham, Safe Cure, Celery Compound, S. S. S., and Tansy wafers. We would be glad to see such an effort succeed, but we question the success very much. In a sheet accompanying the prospectus and addressed to the medical profession, the promoters of the enterprise promise this, in effect: promise to do what the medical press has so greatly censured the religious press for not doing: to expose the pernicious effects of the much-advertised cures; they promise to co-operate with the medical press in its effort to suppress much that is indecent in the advertising department of most newspapers. If they will do that, the RED BACK will extend the glad hand and pat 'em on the back. We will anxiously wait to see some "thoughtful man" "realize himself" on this task.

---

**Wanted**—To purchase a practice in some railroad town in Texas. A good location off railroad not objected to. Please write describing your location, value of practice annually, and number of inhabitants in town. Would buy resident property to get good location. South Texas preferred. Address,

J. R. GEORGE, M. D.,
Corrigan, Texas.

